# Supplementary material for: Coupling Environmental Whole Mixture Toxicity Screening with Unbiased RNA-Seq Reveals Site-Specific Biological Responses in Zebrafish
Source: Toxics. 2023 Feb 21;11(3):201. doi: 10.3390/toxics11030201 (PMC10053777; doi:10.3390/toxics11030201)
Supplement: Supplementary file 1 [file toxics-11-00201-s001.zip › rivermile_supplementary_tables_and_info_final.pdf]

Supplemental information

Paper Title: Coupling environmental whole mixture toxicity screening with unbiased RNAseq reveals site-specific biological responses in zebrafish

Contents:

Figures

Supplementary Figure S1: 1% PHSS extract PAH Concentrations.

Tables

Supplementary Table S1: PAH names and structures included in 33 PAH quantitative method.

Supplementary Table S2: Summary of exposure scenarios and techniques for RNA acquisition and sequencing

Supplementary Table S3: Diagnostic PAH ratios

Supplementary Table S4: Differential Expression of genes named in paper

Supplementary Table S5: Comparison of OPAH gene expression to results from other studies

Data

Available on the supplementary data file

S1: PAH concentrations in PHSS extracts in pg/µL used to determine nominal exposure concentrations.

Data is also available on the Pacific northwest national lab data analytics portal under at:

https://srp.pnnl.gov/samples

sample ids:

6.5W-W-S

RM7W-LFT-W-S FDUP2

RM7W-LFT-W-S FDUP3

RM7W-LFT-W-S FDUP4

S2: The dose response data to each of the PHSD PSD extracts for each endpoint and concentration. Response values are the fraction of fish presenting with the endpoint out of 40 fish.

S3: The differential expression in $\log_2$FC and the P_{adj} for each gene that is differentially expressed in at least one exposure scenario. Differential expressed genes were defined as $|\log_2 FC| > 0.5$ and $P_{adj} < 0.05$.

Supplementary Figure S1: 1% PHSS extract PAH Concentrations.

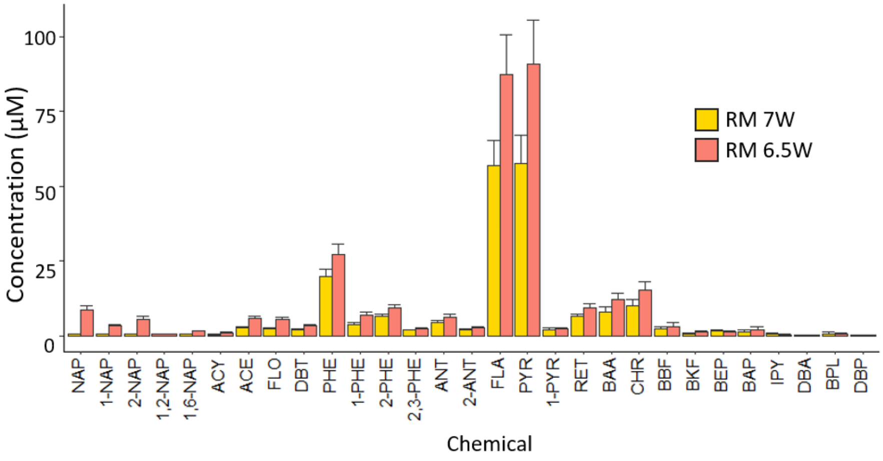

Supplementary Figure S1.

Individual Concentrations of each PAH at or above the LOQ determined by the 33 PAH method for RM 7W and RM 6.5W during Sep 2009 and Jul 2010 respectively. Concentrations report nominal levels in 1 % extract exposures.

Supplementary Table S1: PAHs included in 33 PAH quantitative method: name and structure

Cas Number	Chemical name	abbreviation	structure
573-98-8	1,2-Dimethylnaphthalene	1,2-NAP	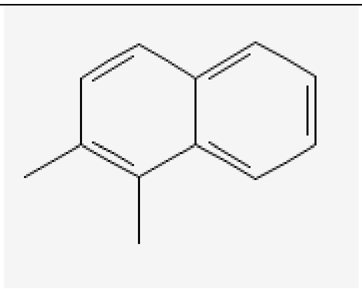
575-43-9	1,6-Dimethylnaphthalene	1,6-NAP	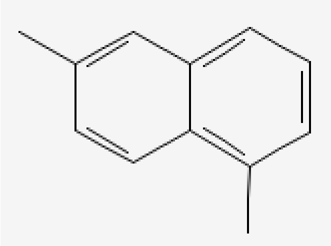
90-12-0	1-Methylnaphthalene	1-NAP	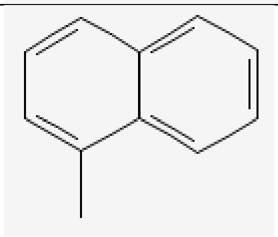
832-69-9	1-Methylphenanthrene	1-PHE	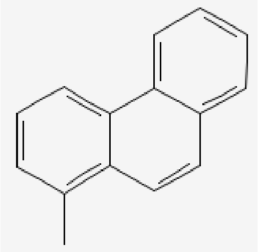
2381-21-7	1-Methylpyrene	1-PYR	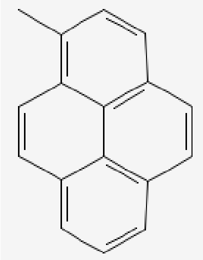
613-06-9	2,3-Dimethylanthracene	2,3-ANT	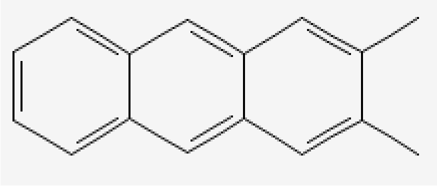

613-12-7	2-Methylanthracene	2-ANT	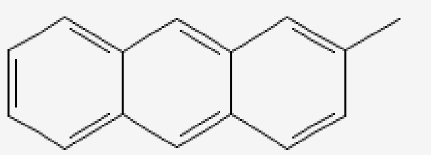
91-57-6	2-Methylnaphthalene	2-NAP	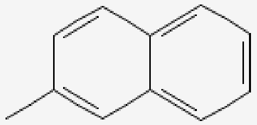
2531-84-2	2-Methylphenanthrene	2-PHE	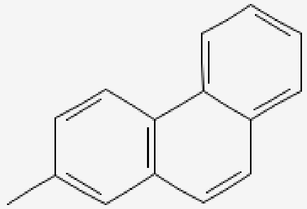
1576-67-6	3,6-Dimethylphenanthrene	2,3-PHE	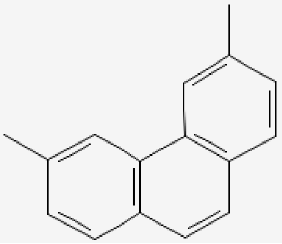
1705-85-7	6-Methylchrysene	6-CHR	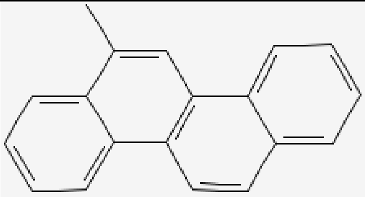
781-43-1	9,10-Dimethylanthracene	9,10-ANT	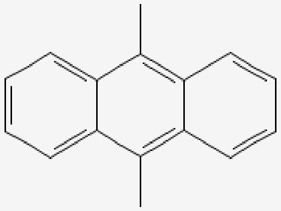
779-02-2	9-Methylanthracene	9-ANT	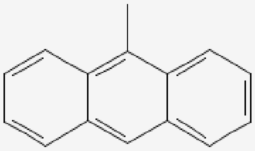
483-65-8	Retene	RET	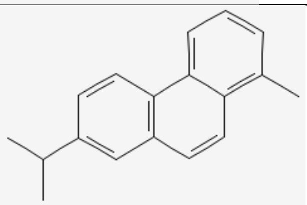

83-32-9	Acenaphthene	ACE	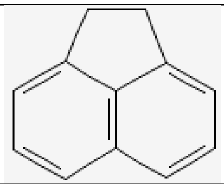
208-96-8	Acenaphthylene	ACY	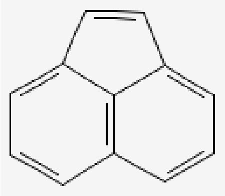
120-12-7	Anthracene	ANT	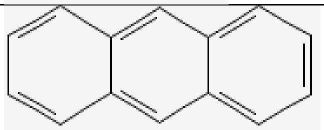
56-55-3	Benz[a]anthracene	BAA	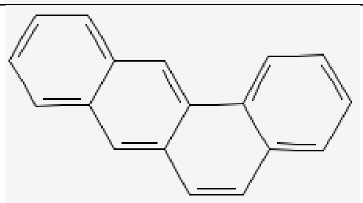
50-32-8	Benzo[a]pyrene	BAP	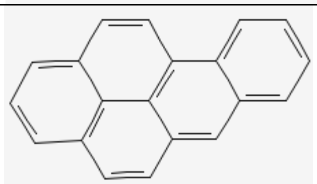
205-99-2	Benzo[b]fluoranthene	BBF	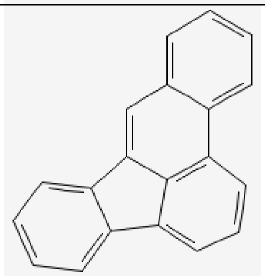
192-97-2	Benzo[e]pyrene	BEP	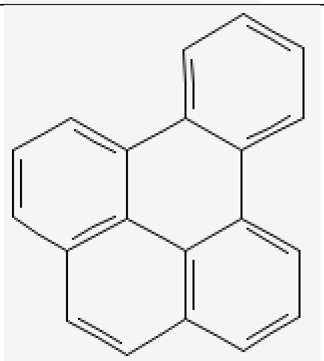

191-24-2	Benzo[g,h,i]perylene	BPL	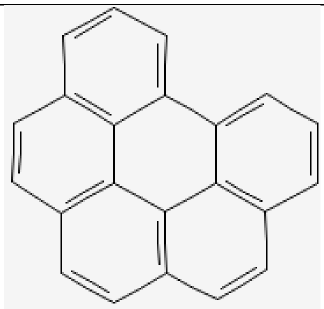
207-08-9	Benzo[k]fluoranthene	BKF	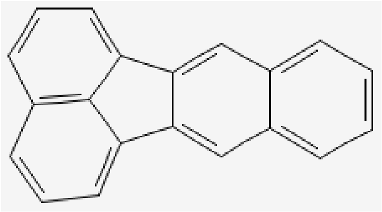
218-01-9	Chrysene	CHR	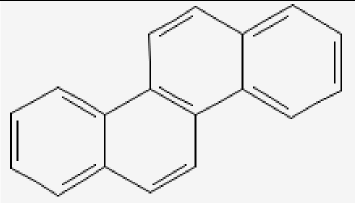
53-70-3	Dibenz[a,h]anthracene	DBA	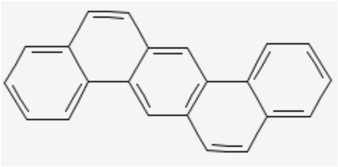
191-30-0	Dibenzo[a,l]pyrene	DBP	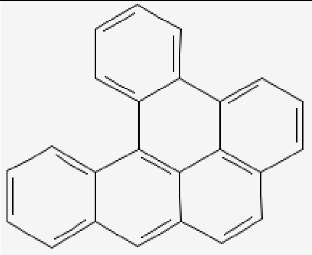
132-65-0	Dibenzothiophene	DBT	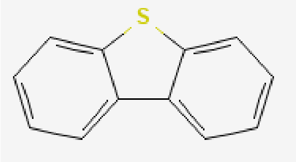
206-44-0	Fluoranthene	FLA	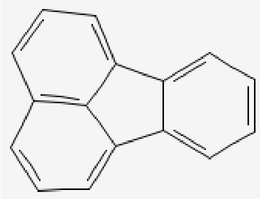
86-73-7	Fluorene	FLO	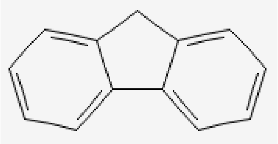

193-39-5	Indeno[1,2,3-cd]pyrene	IPY	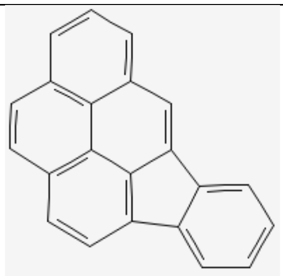
91-20-3	Naphthalene	NAP	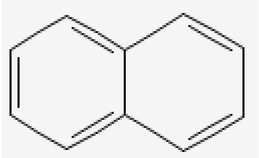
85-01-8	Phenanthrene	PHE	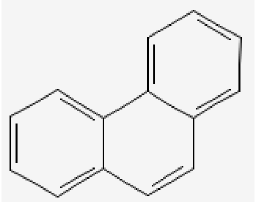
129-00-0	Pyrene	PYR	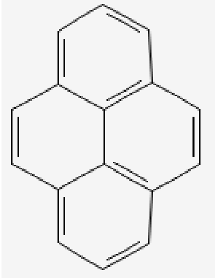

Supplementary Table S2: Summary of exposure scenarios and RNA isolation transcriptional analysis

Chemical	Abbreviation	Control Group	Concentration	Concentration type	RNA Isolation	Library Prep	Seqeuencing Platform	N Samples	N samples final
Benzo[b]fluoranthene	BbF	A	50 uM	EC80	Direct-zol Miniprep	Scriptseq V2	Illumina HISEQ 3000	3	2
Benzo[k]fluoranthene	BkF	A	1.9 uM	EC80	Direct-zol Miniprep	Scriptseq V2	Illumina HISEQ 3000	4	4
Retene	Ret	A	12.2 uM	EC80	Direct-zol Miniprep	Scriptseq V2	Illumina HISEQ 3000	4	4
DMSO	DMSO	A	1%	NA	Direct-zol Miniprep	Scriptseq V2	Illumina HISEQ 3000	3	3
Benz(a)anthracene-7,12-dione	BAAQ	D	10 uM	EC100	Phenolguanadine	PrepX	Illumina HISEQ 2000	3	3
Benzanthrone	BEZO	D	10 uM	EC100	Phenolguanadine	PrepX	Illumina HISEQ 2000	3	3
Phenanthrene-quinone	PHEQ	D	1.2 uM	EC100	Phenolguanadine	PrepX	Illumina HISEQ 2000	3	3
RM 6.5W Jul 2010	RM 6.5W	D	0.75%	NA	Phenolguanadine	PrepX	Illumina HISEQ 2000	3	2
RM 7W Sep 2009	RM 7W	D	0.75%	NA	Phenolguanadine	PrepX	Illumina HISEQ 2000	3	3
DMSO	DMSO	D	1%	NA	Phenolguanadine	PrepX	Illumina HISEQ 2000	3	3

Supplementary Table S3: Diagnostic PAH ratios

chemicals	RM6W ratio	RM7W ratio
FLA/PYR	1.0 ± 0.3	1.0 ± 0.2
RET/CHR	0.6 ± 0.2	0.6 ± 0.2
PHE/ANTH	4 ± 1	5 ± 1

Supplementary Table S4: Differential Expression of genes named in paper

symbol	RIV6	RIV7	Bbf	Bkf	Ret	BEZO	BAAQ	PHEQ
cyp1a	7.92*	7.55*	5.83*	7.82*	7.6*	1.7*	7.63*	0.94*
ptgr1.2	6.34*	5.58*	1	-2.66	2.56	0.54	4.01*	0.68
gstp2	5.39*	5.9*	1.47	1.28	4.34*	2.1*	3.3*	0.68
ahrra	5.36*	5.07*	2.5*	4.78*	5.44*	2.4*	5.32*	-0.95
wfikkn1	5*	4.92*	2.23*	3.7*	4.49*	1.72	4.63*	0.3
cyp1c1	4.63*	4.36*	3.1*	4.02*	5.24*	1.17*	4.88*	0.44
cyp1c2	4.63*	4.17*	2.16*	3.16*	4.61*	0.73*	4.49*	0.7*
ahrrb	4.47*	3.95*	2.63*	4.06*	4.41*	0.65	4.06*	0.01
cyp1b1	4.41*	4.1*	1.49*	3.33*	2.67*	0.17	3.61*	0.58
arf4b	4.25*	3.75*	0.8	-0.59	0.71	0.57	0.74	0.53
nfe2l2b	2.92*	2.35*	0.93*	1.59*	1.08*	1.01*	1.43*	0.02
ugt1ab	2.2*	2.33*	1.17*	1.19*	1.68*	0.71*	1.16*	0.38
foxq1a	1.68*	1.46*	1.71*	3.22*	2.43*	0.11	1.7*	-0.21
tnni1d	-2.18*	-0.17	-1.02	-0.79	-1.02*	-0.06	-0.48	0.13
loxa	-2.24*	-1.35	-0.74	-1.23	-0.85	-1.08	-1.76	-0.31
arr3a	-2.25*	-0.86*	-0.07	0.21	0.31	-1.62*	-0.53	-1.26*
rho	-2.63*	-1.63*	-0.37	0	0	-0.7	-0.3	-1.82*
rom1b	-3.55*	-1.74*	0.24	0.65	-0.05	-1.3	-0.32	-0.87
crygm2d11	-3.74*	-1.42*	-0.06	0.18	-0.54	-3.52*	-0.95	-0.67
crybgx	-4.21*	-1.84*	0.97	0.77	-2.12	-2.18*	-0.64	-1.66
myl2b	-5.72*	-1.01	-1.2	0.52	-1.96	-1.41	-0.62	-0.83
opn1sw1	-6.63*	-2.28*	0.2	-0.68	-0.09	-2.93*	-1.16	-2.47

Gene expression changes called out throughout the study. All values express log_2FCc. Asterisks indicate $p_{adj} \leq 0.05$.

Supplementary Table S5: Comparison of OPAH gene expression to results from other studies

study	present study			Knecht et al. 2013			Misaki et al. 2007
model	developing sebrafish			developing zebrafish			hepg2 cells
technique	RNAseq			qRT_PCR			qRT_PCR
chemical	BEZO (10uM)	BAAQ (uM)	PHEQ (1.2uM)	BEZO (5uM)	BAAQ (5uM)	PHEQ (1uM)	BAAQ
cyp1a	1.76*	7.7*	0.94*	2.5*	9*	1*	4.3*
cyp1b	0.174	3.61*	0.579	<0.5*	4*	0	2*
cyp1c	1.174*	4.883*	0.439	1*	5*	0	
cyp1c2	0.735*	4.487*	0.698*	1*	4*	0	
gpx1a	0.093	1.004*	-0.024	1.27	2.84*	1.01	
gpx7	-0.229	-0.634*	0.021	0.994	1.1	0.95	
gstp1	1.379*	2.401*	0.322	3.16*	12.7*	0.981	
gstp2	2.098*	3.296*	0.679	2.7*	10.3*	0.83	
gclm	0.12	0.533	-0.082	1.53	2.3*	0.906	
nrfx	1.014*	1.43*	0.015	1.06	1.53*	1.37*	
nqo1	-0.066	0.437	0.002	1.09	2.16*	1.55	
hmox	0.569*	0.014	0.104	2.13*	2.03*	3.32*	
akr1c1	1.174*	4.883*	0.439				2.3*
nqo1	-0.066	0.437	0.002				1*

Gene expression data from studies utilizing BEZO, BAAQ, and PHEQ. All expression changes are expressed in $\log_2$FC. Asterisks indicate $p_{adj} \leq 0.05$. Green Shading indicates conserved significance and fold change directionality across studies.
